# Comparative study of whole genome amplification and next generation sequencing performance of single cancer cells

**DOI:** 10.18632/oncotarget.10701

**Published:** 2016-07-19

**Authors:** Anna Babayan, Malik Alawi, Michael Gormley, Volkmar Müller, Harriet Wikman, Ryan P. McMullin, Denis A. Smirnov, Weimin Li, Maria Geffken, Klaus Pantel, Simon A. Joosse

**Affiliations:** ^1^ Department of Tumor Biology, University Medical Center Hamburg-Eppendorf, Hamburg, Germany; ^2^ Bioinformatics Core, University Medical Center Hamburg-Eppendorf, Hamburg, Germany; ^3^ Heinrich-Pette-Institute, Leibniz-Institute for Experimental Virology (HPI), Hamburg, Germany; ^4^ Janssen Research and Development, Spring House, PA, USA; ^5^ Department of Gynecology, University Medical Center Hamburg-Eppendorf, Hamburg, Germany; ^6^ LabConnect LLC, Seattle, WA, USA; ^7^ Department of Transfusion Medicine, University Medical Center Hamburg-Eppendorf, Hamburg, Germany

**Keywords:** NGS, WGA, SNP, allelic dropout, CellSave

## Abstract

**BACKGROUND:**

Whole genome amplification (WGA) is required for single cell genotyping. Effectiveness of currently available WGA technologies in combination with next generation sequencing (NGS) and material preservation is still elusive.

**RESULTS:**

In respect to the accuracy of SNP/mutation, indel, and copy number aberrations (CNA) calling, the HiSeq2000 platform outperformed IonProton in all aspects. Furthermore, more accurate SNP/mutation and indel calling was demonstrated using single tumor cells obtained from EDTA-collected blood in respect to CellSave-preserved blood, whereas CNA analysis in our study was not detectably affected by fixation. Although MDA-based WGA yielded the highest DNA amount, DNA quality was not adequate for downstream analysis. PCR-based WGA demonstrates superiority over MDA-PCR combining technique for SNP and indel analysis in single cells. However, SNP calling performance of MDA-PCR WGA improves with increasing amount of input DNA, whereas CNA analysis does not. The performance of PCR-based WGA did not significantly improve with increase of input material. CNA profiles of single cells, amplified with MDA-PCR technique and sequenced on both HiSeq2000 and IonProton platforms, resembled unamplified DNA the most.

**MATERIALS AND METHODS:**

We analyzed the performance of PCR-based, multiple-displacement amplification (MDA)-based, and MDA-PCR combining WGA techniques (WGA kits Ampli1, REPLI-g, and PicoPlex, respectively) on single and pooled tumor cells obtained from EDTA- and CellSave-preserved blood and archival material. Amplified DNA underwent exome-Seq with the Illumina HiSeq2000 and ThermoFisher IonProton platforms.

**CONCLUSION:**

We demonstrate the feasibility of single cell genotyping of differently preserved material, nevertheless, WGA and NGS approaches have to be chosen carefully depending on the study aims.

## INTRODUCTION

Introduction of single cell analysis led to paradigm shifts in almost all fields of biology and medical sciences as it allows for an accurate representation of the cell-to-cell heterogeneity instead an average measure of an entire cell population [1]. In cancer research, single cell analysis empowers characterization of tumor heterogeneity, and most notably has potential for clinical impact through characterization of circulating tumor cells (CTCs).

CTCs are tumor cells that have separated from primary tumor or current metastases and have infiltrated the systemic blood circulation [2]. Quantification and characterization of CTCs in blood of cancer patients was introduced as a concept of “liquid biopsy”. Enumeration of CTCs as a validated clinical biomarker has been utilized for disease prognosis, diagnosis of minimal residual disease, and monitoring of therapy effectiveness for breast, prostate, and colon cancer [3, 4]. Genomic characterization of CTCs provides insights into genetic heterogeneity of the cancer and metastases and might aid clinical management of cancer patients due to identification of therapy sensitive and resistant clones. Herewith, investigation of single cell genomics may provide the next step towards individualized medicine.

Individual CTCs can be investigated using a combination of whole genome amplification (WGA) and next generation sequencing (NGS) to determine copy number aberrations (CNAs) and gene mutations. However, single cell genomics is associated with certain technical challenges, such as introduction of WGA- and NGS-associated errors. Different technologies for WGA and NGS are currently available but their effectiveness in combination is currently unknown, as well as influence of material preservation on downstream analysis. Suitability of a certain WGA-NGS combination for a particular downstream analysis should be extensively investigated in order to establish a powerful and reliable tool for single cell genomics.

WGA is required for molecular profiling of CTCs since a single cell does not contain enough DNA for direct biomolecular investigation. WGA can be performed by different techniques, such as PCR-based, multiple-displacement amplification (MDA)-based, and a combination of MDA pre-amplification and PCR-amplification. Unlike exponential gain in the first two WGA methods, combined MDA-PCR provides quasi-linear amplification [5–7]. The amplification approach has to be chosen carefully depending on its specific characteristics and the subsequent analysis [8].

An important factor influencing WGA is material preservation, in particular blood preservation. EDTA-preserved blood requires processing as soon as possible [9]. Circulating tumor cells in blood may be preserved in special preservation tubes (CellSave) in order to overcome this requirement. These tubes contain a cell preservative, that stabilizes the sample and maintain cell morphology and cell-surface antigens for up to 96 hours at room temperature, allowing for shipment of the samples. However, fixatives may inhibit DNA amplification, hampering downstream analysis [9, 10]. Most tissue samples are conserved by formalin-fixation, and paraffin-embedding (FFPE), which is difficult to handle in biomolecular analysis due to formalin-induced cross-links [11]. Therefore, it is essential to have WGA methods compatible with these types of preservation.

Downstream analysis of amplified DNA can be performed by massive parallel sequencing using NGS in order to identify SNPs (single nucleotide polymorphisms), indels (insertions-deletions), loss of heterozygosity, structural variations, and CNAs.

Single cell analysis of genomic aberrations by array-CGH is hampered. The necessity of the pre-selected targets’ analysis on template, obtained by random and incomplete genome amplification during WGA [12–14] results in high noise and misinterpretation of the results [15]. Moreover, array-CGH provides limited resolution. The highest resolution for whole genome analysis by array-CGH is 56 kb [16]. In contrast, NGS provides the possibility to examine each nucleotide of the entire amplified product with single base resolution.

Existing NGS platforms differ by library preparation and signal detection methods. Illumina's HiSeq machines exploit sequencing-by-synthesis approach [17, 18]. Currently, HiSeq platforms offer the highest throughput per run, although a sequencing run lasts multiple days [18]. Thermofisher's IonProton sequencers utilize semiconductor sequencing technology, allowing to complete a sequencing run within 4 hours, but homopolymer stretches might be called incorrectly [17].

In this study, we evaluated different protocols, including different methods of preservation, WGA and sequencing to identify an optimal process for single cell sequencing. We compared our findings against unamplified DNA from bulk cell pellets to quantitatively define the impact of different protocols on single cell sequencing. In order to determine the impact of WGA method, we evaluated three different commercially available WGA kits and measured DNA quality and yield. In order to investigate the performance and compatibility of NGS platforms with whole exome sequencing of WGA single cell DNA, we compared the detection of genomic variants (SNPs, indels, and CNAs) from single SK-BR-3 cells spiked and re-captured from EDTA-preserved blood. In order to investigate the influence of material fixatives, we evaluated detection of genomic variants from single SK-BR-3 cells spiked and re-captured from EDTA-preserved vs. CellSave preserved blood. Next, we evaluated the limit of detection and consistency of genomic variant detection with increasing amounts of starting material (i.e. increasing numbers of pooled cells). Finally, we demonstrate proof of principle by evaluating genomic variants detected from CTCs collected from breast cancer patients. Our findings indicate the technical and biological variability in genomic variant detection from single cell sequencing and suggest optimized protocols dependent on starting material and objective (i.e. SNP calling vs. CNA calling).

## RESULTS

### Whole genome amplification of single cells

Three WGA kits (i.e. Ampli1, PicoPlex, and REPLI-g representing PCR-based, combined MDA-PCR, and MDA-based WGA technique, respectively) were used to amplify single cell samples of 4 groups: A) 10 individual SK-BR-3 cells spiked and picked from EDTA-preserved blood; B) 10 individual SK-BR-3 cells spiked and picked from CellSave-preserved blood; C) 10 single SK-BR-3 cells picked from FFPE SK-BR-3 cells; and D) 10 individual CTCs picked from EDTA-collected blood of breast cancer patients. In total, 120 single cells were individually processed by WGA. DNA yield and success rate, as measured by multiplex PCR of *GAPDH* gene, of the tested WGA kits are presented in Table 1 and on Supplementary Figure S3.

**Table 1:** Mean DNA yield (Table 1A) and PCR quality control success rate (Table 1B) for single SK-BR-3 cells and CTCs extracted from EDTA and CellSave preservation tubes, and FFPE material, after amplification with Ampli1, PicoPlex, and REPLI-g WGA kits

Table 1A: DNA output, μg					
WGA kit	WGA output, mean ± st. dev., μg				
	SK-BR-3 EDTA	SK-BR-3 CellSave	SK-BR-3 FFPE	CTC EDTA	Average
**Ampli1**	7.067 ± 1.082	5.857 ± 2.226	6.738 ± 1.608	4.688 ± 3.187	6.088 ± 2.285
**PicoPlex**	2.864 ± 1.137	3.392 ± 2.320	4.710 ± 0.406	4.013 ± 1.236	3.745 ± 1.555
**REPLI-g**	15.394 ± 1.353	11.374 ± 1.252	77.966 ± 30.820	31.410 ± 12.841	34.036 ± 31.232

Table 1B: PCR quality control success rate, %					
WGA kit	PCR quality control success rate, %				
	SK-BR-3 EDTA	SK-BR-3 CellSave	SK-BR-3 FFPE	CTC EDTA	Average
**Ampli1**	100	100	100	70	93
**PicoPlex**	80	100	100	100	95
**REPLI-g**	70	70	30	30	50

CTC – circulating tumor cell; st.dev – standard deviation.

PCR-based WGA (Ampli1 kit) demonstrated an average DNA yield of 7.07 μg, 5.86 μg, 6.74 μg and 4.69 μg for the 4 different 10-sample sets respectively with the average DNA yield 6.09 μg (Table 1A). The *GAPDH* multiplex-PCR demonstrated a 100% success rate for the experiment with EDTA tubes, CellSave tubes, and FFPE experiments, whereas the amplification of the patients’ CTCs demonstrated a success of 70% for CTCs (Table 1B). The average DNA yield for MDA-PCR WGA (PicoPlex kit) was 2.86 μg, 3.39 μg, 4.71 μg and 4.01 μg for the 4 different 10-sample sets respectively and 3.74 μg on average for all 40 samples. Quality control PCR demonstrated 100% success rate in all groups except single SK-BR-3 cells picked from EDTA blood (80% success rate). The MDA-based WGA (REPLI-g kit) demonstrated the highest DNA yield: 15.39 μg, 11.37 μg, 77.97 μg and 31.41 μg in the same 4 experimental groups respectively. The average DNA output was 34.04 μg for all 40 samples. Quality control PCR demonstrated 70% success rate in cases of single SK-BR-3 picked from EDTA and CellSave tubes and 30% in cases of FFPE SK-BR-3 cells as well as patient CTCs. Among all tested WGA kits MDA-based WGA demonstrated the highest DNA yield along all sample group, however with the lowest success rate (50% average). PCR-based and MDA-PCR WGA techniques demonstrated comparable success rates (on average 93 and 95%, respectively) with DNA yield prevalence of PCR-based WGA over samples processed with MDA-PCR WGA technique in all compared groups (on average 6.09 and 3.74 μg, respectively).

### SNP/mutation, indel, and CNA analyses of SK-BR-3 cells, obtained from EDTA-preserved blood

**Figure 1:**
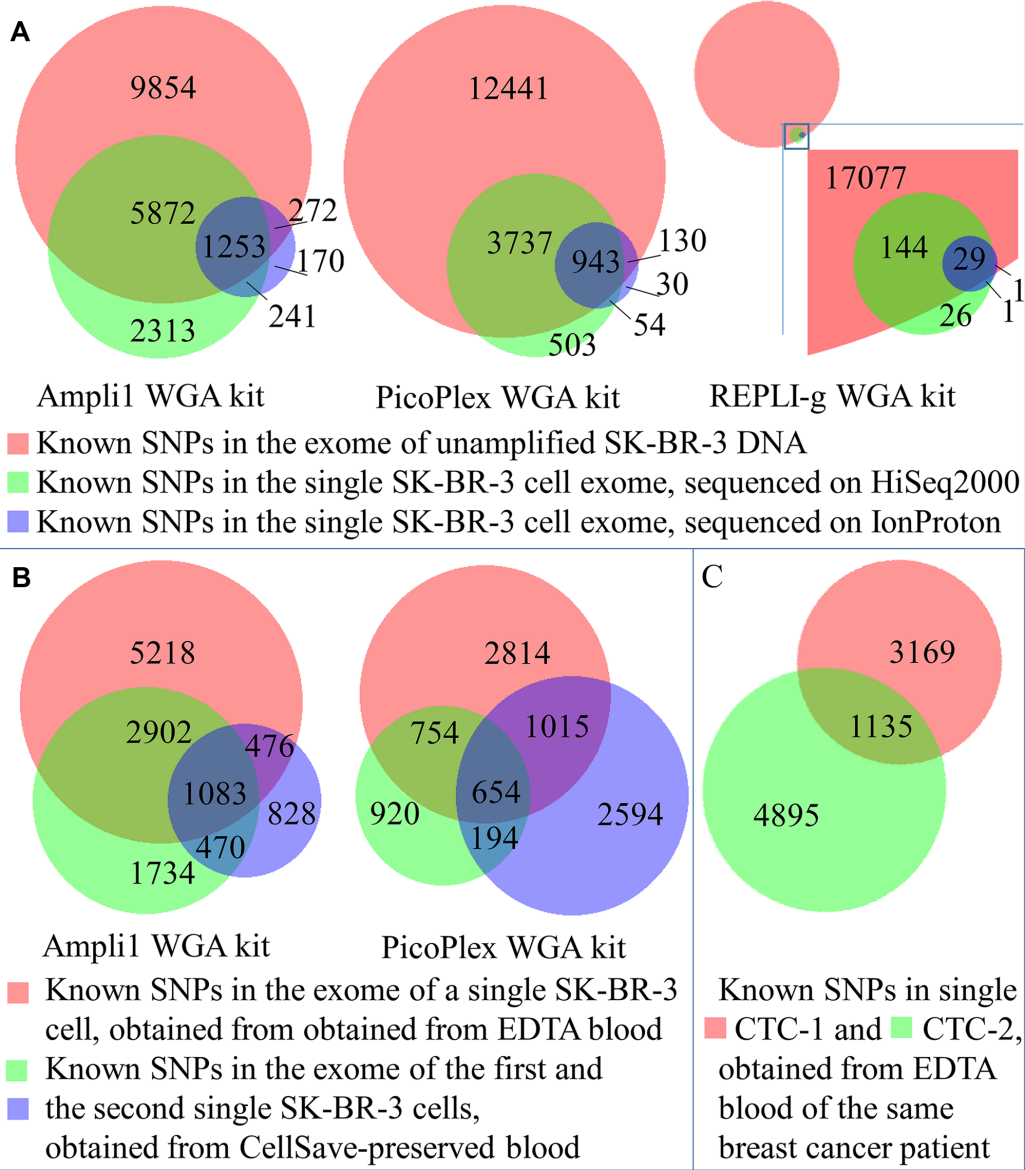
Distribution of identified known SNPs between datasets (**A**) Known SNPs identified in single cells, amplified with Ampli1, PicoPlex, and REPLI-g WGA kits and obtained from EDTA-preserved blood in comparison to unamplified DNA. (**B**) Known SNPs identified in single cells, amplified with Ampli1 or PicoPlex and obtained from EDTA- and CellSave-preserved blood in comparison to unamplified DNA from unfixed cells. (**C**) Known SNPs identified in single CTCs, amplified with PicoPlex in comparison to each other.

Genomic variants detected from single cells recovered from EDTA-preserved blood were analyzed to compare sequencing platforms and WGA methods. Variants detected in single cell analyses were compared to variants detected in bulk cell pellets without WGA as a gold standard. We report sequencing quality statistics (e.g. read depth), the total number of single nucleotide variants (SNVs) and indels detected, including both previously reported SNPs and indels and novel variants, the allelic dropout rate and the sensitivity and positive predictive value (PPV) of detection compared against unamplified DNA as metrics to compare different protocols. Sequencing with HiSeq2000 platform produced more reads and provided higher depth and breadth of target base coverage, higher mapping rates, and lower duplicate rates compared to IonProton. Comparing the applied WGA procedures, the highest numbers of clean reads, mapping and duplicate rates were observed for MDA-based WGA kit. The complete characteristics of NGS data are presented in Supplementary Table S1. The number of total and known SNPs identified with HiSeq2000 platform was higher than for IonProton regardless of the WGA method used (Figure 1A). Sequencing with the HiSeq2000 platform resulted in 7125, 4680, 173 known SNPs detected with PCR-based, MDA-PCR, and MDA-based WGA techniques, respectively, and concordant with known SNPs detected in bulk unamplified DNA. Sequencing with the IonProton platform resulted in the detection of 1525, 1073, and 30 concordant known SNPs with respective WGA kits. Sensitivity, the probability of detecting a known SNP found in the reference sample in the single cell samples, was also higher in samples sequenced with HiSeq2000 with 41.3, 27.1% and 1.0% for PCR-based, MDA-PCR, and MDA-based WGA experiments, respectively (Table 2).

**Table 2:** The counts and statistics of SNP and indel calls in SK-BR-3 individual cells, obtained from EDTA-collected blood, amplified with Ampli1, PicoPlex, and REPLI-g WGA kits and sequenced with Illumina's HiSeq2000 and ThermoFisher's IonProton NGS platforms

Groups	WGA kit	Ampli1		PicoPlex		REPLI-g		Reference exome
	NGS platform	HiSeq2000	IonProton	HiSeq2000	IonProton	HiSeq2000	IonProton	HiSeq2000
**SNP statistics**	Total SNPs	9944	1986	9948	1695	403	64	17659
	Known SNP	9679	1936	5237	1157	200	31	17251
	Known SNP, %	97.3	97.5	52.6	68.3	49.6	48.4	97.7
	SNP novel	265	50	4711	538	203	33	408
	ADO, %	9.0	19.8	24.0	42.4	100.0	100.0	na
	Common SNPs with known SNPs in reference	7125	1525	4680	1073	173	30	17251
	Sensitivity, %	41.3	8.8	27.1	6.2	1.0	0.2	100.0
	PPV, %	73.6	78.8	89.4	92.7	86.5	96.8	na
**Indel statistics**	Total indels	1148	2688	2469	1688	140	52	502
	Known indels	176	23	82	14	3	1	310
	Known indels, %	15.3	0.9	3.3	0.8	2.1	1.9	61.8
	Common indels with known indels in reference	116	16	71	11	2	1	310
	Sensitivity, %	37.4	5.2	22.9	3.6	0.7	0.3	100.0
	PPV, %	65.9	69.6	86.6	78.6	66.7	100.0	na
**CNA analysis**	Spearman correlation coefficient (r)	0.66	0.63	0.81	0.80	0.25	0.25	na
	*P*-value	<0.001	<0.001	<0.001	<0.001	<0.001	<0.001	na

Total SNPs/indels – number of all identified SNPs/indels. Known SNP – fraction of SNPs/indels, present in SNP database. Novel SNPs/indels – number of SNPs/indels, not present in SNP database. ADO – allelic dropout. PPV – positive predictive value.

**Figure 2:**
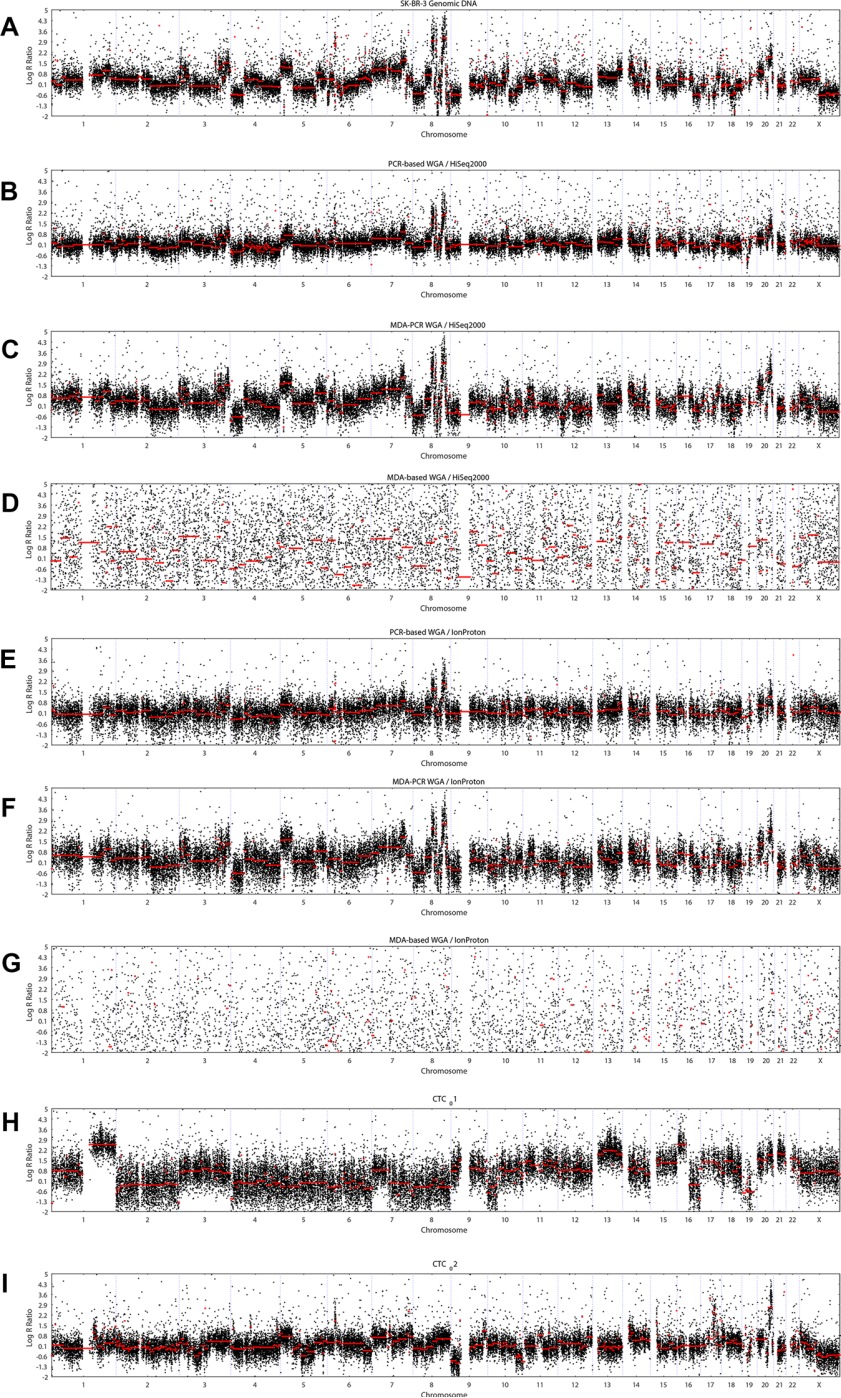
Plots of CNA profiles along the whole genome (x axis) (**A**) CNA profile of unamplified DNA from unfixed cells. (**B**–**G**) plots of CNAs in single SK-BR-3 cells, obtained from EDTA-preserved blood. (**H**, **I**) CNA profiles of individual CTCs, obtained from EDTA-preserved blood of the same breast cancer patient. WGA kits: (B, E) Ampli1; (C, F, H, I) PicoPlex; (D, G) REPLI-g.

Novel SNVs were identified in single cells, as well as in genomic DNA, which might be sequencing or amplification errors. Higher numbers of novel SNVs were observed for HiSeq2000 over IonProton sequenced samples (265 vs 50, 4711 vs 538, and 203 vs 33 for PCR-based, MDA-PCR, and MDA-based WGA kits and HiSeq2000 vs IonProton NGS, respectively, in comparison to 408 novel SNVs detected in SK-BR-3 genomic DNA). The highest number of novel SNPs was observed for the cell amplified with combined MDA-PCR technique and HiSeq2000-sequenced (4711 SNPs). Among the samples sequenced with the same NGS platform, more known indels were identified in samples amplified with PCR-based WGA (176 vs 23, 82 vs 14, and 3 vs 1 for PCR-based, MDA-PCR, and MDA-based WGA kits compared for HiSeq2000 vs IonProton, respectively). The fraction of known indels was the highest for PCR-based WGA-HiSeq2000 analysis (15.3%) (Table 2). CNA profiles from single cells were compared with the CNA profile of genomic SK-BR-3 DNA using Spearman correlation (Figure 2A–2G). Correlation between whole genome amplified single cells and genomic DNA did not depend on NGS platform, but was dependent on WGA kit. The cells amplified with PCR-based, MDA-PCR, and MDA-based WGA techniques demonstrated median (*r* < 0.7), strong (*r* > 0.8) and weak (*r* < 0.3) correlation with genomic DNA, respectively (Table 2).

Allelic dropout (ADO) rates demonstrated dependence on both WGA and sequencing platform (Table 2). ADO rates were lower in HiSeq2000-sequenced samples in comparison to IonProton with outperformance of the PCR-based WGA technique within the same NGS platform (9, 24, and 100% for cells, amplified with PCR-based, MDA-PCR, and MDA-based WGA kits and sequenced on Hiseq200 vs 20, 42, and 100% in IonProton group, respectively). Based on the obtained results of the first NGS experiments, we excluded MDA-based WGA technique (REPLI-g kit) and IonProton platform from further analyses.

### SNP/mutation, indel, and CNA analyses of single and pooled SK-BR-3 cells, obtained from CellSave-preserved blood in comparison to single cells from EDTA-collected blood

To investigate the detection limit with increasing amount of starting material for WGA, as well as the influence of CellSave preservative on WGA and NGS performance we analyzed duplicates of 1, 3, 5, and 10 pooled SK-BR-3 cells amplified with PCR-based and MDA-PCR combined WGA techniques and sequenced on Illumina's Hiseq2000. The whole obtained data is presented in Supplementary Table S1.

Comparison between single cells obtained from EDTA- and CellSave-collected blood revealed lower numbers of the total and known identified SNPs and indels, and higher number of novel SNPs and indels in cells from CellSave-preserved blood. Sensitivity of SNP and indel calling was lower for single cells from CellSave tubes in comparison to single cells obtained from EDTA-preserved blood (Table 3). The overlap in known SNPs detected from single cells in EDTA and CellSave preserved blood was similar to the overlap detected from technical replicates of single cells in CellSave preserved blood (Figure 1B), indicating that technical bias from other sources is greater than variation from the preservation method. As described above, comparison of findings obtained by different WGA kits demonstrates superiority of PCR-based WGA technique over MDA-PCR combined WGA technique for SNP and indel analysis in single cells, as indicated by the higher sensitivity of PCR-based WGA (Table 3).

**Table 3:** The counts and statistics of SNP and indel calls in single SK-BR-3 cells, analyzed in duplicates, obtained from CellSave-preserved blood, in comparison to single SK-BR-3 cells, obtained from EDTA-collected blood

Groups of experiments	WGA kit	Ampli1			PicoPlex			SK-BR-3 genomic DNA
	NGS	HiSeq2000			HiSeq2000			HiSeq2000
	Material preservation	EDTA	CellSave	CellSave	EDTA	CellSave	CellSave	na
	Number of cells	1	1	1	1	1	1	∼8×10^6^
**SNP statistics**	Total SNPs	9944	7826	4088	9948	9738	9821	17659
	Known SNP	9676	6189	2857	5237	2522	4457	17251
	Known SNP, %	97.3	79.1	69.9	52.6	25.9	45.4	97.7
	SNP novel	265	1637	1231	4711	7216	5364	408
	ADO rate, %	9.0	36.4	78.5	54.0	74.3	66.4	na
	Common SNPs with known SNPs in reference	7125	5680	2381	4680	2088	2885	17251
	Sensitivity, %	41.3	32.9	13.8	27.1	12.1	16.7	na
	PPV, %	73.6	91.8	83.3	89.4	82.8	64.7	na
**Indel statistics**	Total indels	1148	723	165	2469	790	914	502
	Known indels	176	89	36	82	24	63	310
	Known indels, %	15.3	12.3	21.8	3.3	3.0	6.9	61.8
	Common indels with known indels in reference	116	76	32	71	19	42	310
	Sensitivity, %	37.4	24.5	10.3	22.9	6.1	13.6	na
	PPV, %	65.9	85.4	88.9	86.6	79.2	66.7	na
**CNA analysis**	Spearman correlation coefficient (r)	0.64	0.84	0.25	0.81	0.69	0.09	na
	*P*-value	<0.001	<0.001	<0.001	<0.001	<0.001	<0.001	na

Total SNPs/indels – number of all identified SNPs/indels. Known SNP – fraction of SNPs/indels, present in SNP database. Novel SNPs/indels – number of SNPs/indels, not present in SNP database. ADO – allelic dropout. PPV – positive predictive value.

**Figure 3:**
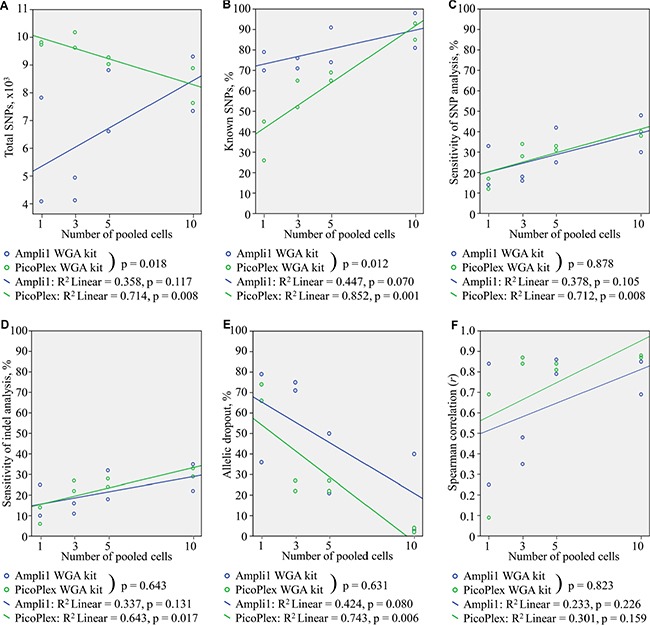
Characteristics of pooled 1, 3, 5, and 10 SK-BR-3 cells, obtained from CellSave-preserved blood, amplified with Ampli1 and PicoPlex WGA kits, and sequenced with HiSeq2000 NGS platform (**A**) Total identified SNPs. (**B**) Known identified SNPs. (**C**) Concordance of identified SNPs with reference dataset. (**D**) Sensitivity of the SNP calling analysis. (**E**) Allelic dropout. (**F**) Correlation of CNA profiles with CNA profile of unamplified DNA.

Analyses of pooled cells demonstrated that the numbers of the identified total SNPs/mutations increased with increasing number of pooled cells for experiments with PCR-based WGA and decreased for experiments with MDA-PCR WGA technique, statistically significant for MDA-PCR experiments only. In contrast, the numbers of the identified known SNPs/mutations increased with increasing number of pooled cells for both WGA kits, however statistically significant between different groups of pooled cells for MDA-PCR only. Moreover, the rate of change of detection of total and known SNPs with increasing number of cells was found to be different with PCR-based and MDA-PCR combined WGA techniques. PCR-based WGA technique appears to have more variability in performance as indicated by the variance in the number of total SNPs detected (Figure 3A). Variance is smaller in the percentage of known SNPs detected (Figure 3B). In addition, the increase in the percentage of known SNPs detected with increasing numbers of pooled cells is greater for the MDA-PCR combined WGA technique (Figure 3A, 3B).

Sensitivity of SNP and indel analyses increased with increasing number of pooled cells. The effect was statistically significant for MDA-PCR, but not PCR-based WGA as indicated by significant correlation. However, the differences in kits’ performance were not significantly different by these metrics (Figure 3C, 3D).

Samples analyzed with either kit showed similar ADO rates (3–79 and 2–74%, respectively) (Supplementary Table S1). With each kit, ADO rates decreased with increasing numbers of pooled cells, indicating that the high ADO rate with single cells is largely attributed to WGA. This effect was significantly different for WGA with MDA-PCR only (Figure 3E).

Correlation between CNA profiles of genomic DNA and analyzed samples increased along with the number of pooled cells for both WGA kits, however, this effect was not statistically significant. There was no significant difference in the rate of change of performance between the two kits (Figure 3F).

The obtained results suggest that the PCR-based WGA technique is superior to the MDA-PCR technique for SNP and indel analysis in single cells (Table 3). Notably, detection of SNPs by MDA-PCR WGA significantly improves with the number of pooled cells (i.e. increasing amount of input DNA). The performance of PCR-based WGA did not significantly improve with increase of input material in any case. This suggests a greater effect of WGA for MDA-PCR amplification in comparison to PCR-based amplification.

### Genomic characterization of patient tumor cells

As proof of principle, two CTCs from a metastatic breast cancer patient with primary metastatic disease, ER-positive and HER2-negative, were isolated from 10 ml of blood obtained in an EDTA tube. The MDA-PCR WGA technology was used to amplify the genomes of the individual cells, followed by exome sequencing using the HiSeq2000 platform. MDA-PCR WGA technology was chosen based on its performance in CNA analysis (Table 2). The subsequent CNA analysis demonstrated two genetically different profiles (Figure 2H–2I), suggesting cancer genetic heterogeneity of this patient's disease. Both CTCs carry gain of chromosome 1q, which has been identified previously as an universal genomic feature of breast cancer [19]. Additionally, CTC-1 demonstrates copy number variation typical for luminal breast cancer including chromosome 16p gain and chromosome 16q loss. In contrast, CTC-2 is strongly characterized by chromosome 9p loss. SNP calling analysis revealed 1135 SNPs and 15 indels common in both cells (Figure 1C). Mutation analysis revealed 5 missense mutations annotated in COSMIC database [20]. Mutations in genes *CHEK2*, *PRAME*, and *KIT* were present in both CTCs, mutation in gene *FGFR2* was detected in CTC-1 only and in gene *TP53* – in CTC-2 only (Table 4).

**Table 4:** The counts and statistics of SNP and indel calls in CTCs

Groups	Cell	CTC-1	CTC-2
	WGA kit	PicoPlex	
	NGS	HiSeq2000	
	Blood preservative	EDTA	
**SNP statistics and concordance between CTCs**	Total SNPs	34994	14658
	Known SNP	4304	6030
	Known SNP, %	12.3	41.1
	SNP novel	30690	8628
	Common in both datasets known SNPs	1135	
	Fraction of common known from known identified in dataset, %	26.4	18.8
**Indel statistics and concordance between CTCs**	Total indels	4383	4103
	Known indels	42	81
	Known indels, %	1.0	2.0
	Common in both datasets known indels	15	
	Fraction of common known from known identified in dataset, %	37.7	18.5
**CNA analysis**	Correlation between CTCs, r	0.10	
**Mutation analysis (amino acid change)**	Present in both CTCs	*CHEK2* (K373E)	
		*KIT* (M541L)	
		*PRAME* (W7R)	
	Present in only one CTC	*FGFR2* (Y376C)	wt
		wt	*TP53* (E285K)

Total SNPs/indels – number of all identified SNPs/indels. Known SNP – fraction of SNPs/indels, present in SNP database. Novel SNPs – number of SNPs, not present in SNP database. ADO – allelic dropout. r – Spearman correlation coefficient. wt – wild type.

## DISCUSSION

In the study presented here, the performance of single cell WGA and subsequent whole exome sequencing were investigated on 2 different NGS platforms. Illumina's HiSeq platforms are widely used in human genome research due to their accuracy. Sequencing with ThermoFisher's IonProton can be faster and more cost-effective per run, however, sequencing with IonProton may result in substantial decrease of effective coverage depth due to the high abundancy of PCR and optical duplicates. Emulsion PCR, utilized for library preparation in IonProton technology, is thought to be the main source of PCR duplicates [21]. Moreover, the introduction of indels is a well-documented disadvantage of the semiconductor sequencing utilized in IonProton [17]. Nevertheless, our study shows that CNA analysis was not affected by the described disadvantages of semiconductor sequencing and demonstrated comparable results for samples sequenced on both NGS platforms.

Important applications of NGS such as SNP/mutation, indel, and CNA calling seem to be especially hampered in single cell analysis due to relatively high variance in amplification efficiency across the genome as a result of WGA [6, 12, 13]. Allelic dropout (ADO), defined as the complete absence of one allele of heterozygous loci, is one of the major concerns associated with WGA, leading to false interpretation of SNP/mutation and indel calling results. In our study, PCR-based WGA technique demonstrated lower ADO rates on the single cell level and consequently more accurate SNP/mutation and indel calling independent of sequencing platform, blood preservative, and number of pooled cells. These data suggest that PCR-based WGA (Ampli1 kit) outperforms MDA-PCR combining (PicoPlex kit) and MDA-based WGA techniques (REPLI-g kit) for SNP/mutations and indel calling. (Table 2).

The differences in the amplification approach and source material might explain the reduction of SNP calling sensitivity in MDA-processed samples. We used single cells fixed with 0.5% paraformaldehyde for WGA. Phi29 polymerase, utilized in the MDA-based WGA approach, demonstrates low efficiency when used with fragmented and/or cross-linked DNA since it requires average genomic DNA fragment sizes of approximately 2 kb in order to amplify DNA without introducing any bias [22, 23]. PCR-based methods are generally more tolerant to damaged DNA, explaining a better efficiency for the PCR-based approach then the MDA-based WGA.

However, adaptor-ligation PCR, utilized in some PCR-based WGA kits (e.g., Ampli1), has certain limitations. Site-specific digestion of template DNA prior to PCR by the MseI enzyme [24] results in a wide distribution of fragment lengths. In silico analysis (data not shown) demonstrates that only 38% of 19 × 10^6^ fragments produced by MseI restriction of the human genome have length 100–500 bp and therefore sufficient for exome-capturing and size-selection for library preparation. In order to optimize single cell sequencing, revision of the current exome capturing regions is required.

Commercially available exome enrichment kits have not been optimized for WGA products. The usage of fragmented WGA DNA as template might drastically reduce capturing efficacy. Moreover, a significant fraction of template DNA can be nonspecifically enriched outside target regions, varying from kit to kit [25–27], causing identification of thousands of high quality SNPs outside the target regions [25]. A limitation of this study is that only one exome capturing kit has been tested and thus, it cannot be ruled out that other capturing kits may have different results. Exome capturing in which smaller regions are targeted might outperform capturing of larger genomic regions. Another limitation of this study is that we used SK-BR-3 bulk DNA, sequenced on HiSeq2000 as reference for SNP/mutation, indel and CNA analysis for SK-BR-3 cells, sequenced on both HiSeq2000 and IonProton platforms. IonProton sequenced bulk SK-BR-3 DNA used as reference might improve results of IonProton-sequenced samples.

Although samples amplified with MDA-based WGA technique (REPLI-g kit) demonstrated the highest DNA yield from a single cell, the quality of the obtained DNA was remarkably low and insufficient for appropriate SNP/mutation, indel, and CNA analyses. Based on our experience and observations of de Bourcy et al. [8] and Bergen et al. [28], we conclude that input of at least 10 ng of genomic DNA and tailoring of the MDA reaction to obtain just enough DNA for further analysis is a key to optimal MDA performance. Further biases in MDA-based WGA can distort CNA analysis and have been described elsewhere, these include uneven representation and non-specific amplification of the genome, a large variability in amplification bias among the products, chimera formation, and dislocated sequences [8, 29–31].

Single cells from EDTA-collected blood demonstrated higher sensitivity for SNP/mutation and indel analyses, than single cells from CellSave-preserved blood. Since EDTA-collected blood requires timely processing after collection [9], CellSave blood preservation could be of great value in e.g., multicenter studies. In this study, we examined the consistency of SNP/mutation and indel calling performance in 1, 3, 5, and 10 pooled cells in comparison to unamplified genomic DNA and the influence of WGA technique on the results. SNP/mutation and indel analyses of single and pooled cells revealed high variability in results for PCR-based and MDA-PCR combining WGA techniques on single cell level, decreasing with the number of pooled cells.

The concordance of the identified SNPs/mutations in 1, 3, 5 and 10 cells from CellSave-preserved blood with the reference was invariant to the WGA technique used and improved with increasing number of pooled cells. For MDA-PCR amplified DNA, the sensitivity $\left(\frac{\text{true}-\text{positives}}{\text{true}-\text{positive}+\text{false}-\text{negatives}}\right)$ of the SNP/mutation analysis increases with increasing number of pooled cells, in association with a decrease in ADO rates. A similar trend was detected with DNA amplified with the PCR-based WGA technique, although the effects were not significant. The effects may have been obscured by the relatively high variance observed with PCR-based amplification. Similarly, CNAs detected from single or pooled cells demonstrates a trend for increasing correlation with calls made from unamplified DNA. These effects are not significant which may be due to the relatively high variance in correlation with low number of starting cells (Figure 3).

CNA profiles of single cells from EDTA-collected blood demonstrated higher correlation with unamplified DNA than single cells from CellSave-preserved blood. Thus, CNA profiles of even a single cell from EDTA-collected blood, amplified with MDA-PCR combining WGA technique and sequenced on both HiSeq2000 and IonProton, demonstrated strong correlation (*r* ≥ 0.8) with unamplified DNA (Table 2) in contrast to single cells from CellSave tube amplified with MDA-PCR WGA technique (*r* = 0.1 and *r* = 0.7) (Table 3). The experiments with PCR-based WGA demonstrated moderate correlation between CNA profiles of unamplified DNA and DNA amplified from EDTA-preserved single cells (*r* < 0.7) (Table 2), whereas CNA detected in cells from CellSave tube demonstrated correlation of *r* = 0.3 and *r* = 0.8 (Table 3). Moreover, as few as 3 pooled cells from CellSave-preserved blood resembled CNA pattern of unamplified DNA with strong correlation, whereas samples amplified with PCR-based WGA reached the same correlation level with 5 pooled cells (Supplementary Table S1).

A recent study from our lab has demonstrated genetic heterogeneity within a cancer cell line upon sequencing single cells [32]. Therefore, it cannot be ruled out that the low concordance of SNP/mutation calling between single cells might also be the effect of heterogeneity in addition to WGA artifacts. However, the strong correlations of CNA of the SK-BR-3 cell-line between different lineages published in the past [11] suggests that its overall genome is relatively stable. Further research entailing deep sequencing of unamplified genomic DNA will reveal the genetic heterogeneity of this cell line. It has been noted that WGA strongly affects CNA analysis due to imbalanced amplification of alleles [5, 13]. Moreover, non-linear amplification is random and is not reproducible for the same DNA template [14]. Although CNA analysis does not require exome capturing and is possible on whole genome shallow sequenced data, we performed CNA analysis on whole exome sequencing data and demonstrated that the quality of the obtained DNA by both PCR-based and MDA-PCR combined WGA techniques was adequate for qualitative assessment of CNA patterns. Deeper exome sequencing may compensate imbalanced allele amplification, crucial for CNA analysis of shallow sequenced whole genome data.

Sequencing CTCs from cancer patients has been suggested as a “liquid biopsy” that could be used to study tumor heterogeneity and find therapy associated markers [33]. In our study, we identified 3 cancer-associated mutations, 1135 SNPs, and 15 indels common in two CTCs from a single breast cancer patient, however their CNA profiles were not similar, reflecting intra-patient heterogeneity. Given the findings presented from our benchmarking analyses, it is difficult to separate true biological variants from variation introduced by WGA or sequencing artifacts. However, identification of non-overlapping mutations in *FGFR2* and *TP53* genes might indicate clonal evolution of the tumor. Further single cell genomic research and improved WGA methods may enable us to investigate cancer evolution during tumor development and under therapy pressure leading to treatment resistance using CTC sequencing.

## MATERIALS AND METHODS

### Experimental design

First, we investigated performance of 3 WGA kits, representing 3 WGA methods, in 4 groups of source material, differing by origin and preservation method. The 4 sources of material included: A) individual SK-BR-3 cells obtained from EDTA-preserved blood; B) individual SK-BR-3 cells obtained from CellSave-preserved blood; C) single SK-BR-3 cells picked from FFPE SK-BR-3 cells; and D) individual CTCs obtained from EDTA-preserved blood from a breast cancer patient.

WGA was performed using PCR-based Ampli1 (WG-001-050-R02, SiliconBiosystems), combined MDA-PCR PicoPlex (E2620L, NewEngland Biolabs,), and MDA-based REPLI-g (150343, Qiagen) WGA kits according to the manufacturers’ recommendations. After DNA yield and quality per WGA kit were estimated, DNA of single cells from each WGA group was used for whole exome NGS on 2 platforms. Briefly, 3 SK-BR-3 cells, obtained from EDTA-preserved blood and amplified with Ampli1, PicoPlex, and REPLI-g kit, were analyzed with both HiSeq200 and IonProton platforms.

Based on results obtained from initial pilot experiments, the IonProton platform and Repli-G WGA kit were excluded from further experiments. The second round of experiments included WGA of single and pooled cells in duplicates and NGS of obtained DNA in order to investigate the performance and the limit of detection with increasing amounts of material. Duplicates of 1, 3, 5, and 10 pooled SK-BR-3 cells obtained from CellSave-preserved blood and amplified with Ampli1 and PicoPlex kits were sequenced on HiSeq2000.

Subsequently, a proof of principle experiment was performed on 2 individual CTCs obtained from EDTA-collected blood of a breast cancer patient. The cells were individually amplified with PicoPlex WGA kit and sequenced on HiSeq2000 (Supplementary Figure S1). In total, 120 single cells and 72 pooled cells were processed.

### Cell culture

The breast cancer cell line SK-BR-3 was acquired from ATCC and cultivated under prescribed conditions. The cells were harvested using trypsin/EDTA (R001100; Gibco), washed and resuspended in PBS (14190-094; Gibco) for further experiments. Genomic DNA was extracted using the Blood&Cell Culture DNA Mini Kit (13323, Qiagen). The same cell line was previously formalin-fixed, paraffin-embedded, and stored for over 3 years to simulate archival material.

### Blood sampling

Blood from healthy individuals and metastatic breast cancer patients was obtained from the Department of Transfusion Medicine and Department of Gynecology at the University Medical Center Hamburg-Eppendorf, respectively. All study participants gave written informed consent. The examination of blood from breast cancer patients was approved by the local ethics review board Aerztekammer Hamburg (OB/V/03). Breast cancer patients’ blood was sampled in EDTA collection tubes (01.1605.001, Sarstedt). Blood from healthy donors was collected either in EDTA or CellSave tubes (7900005, Janssen Diagnostics) and spiked with SK-BR-3 cells to simulate CTCs.

### Patient data

The patient who received her CTCs analyzed by NGS was diagnosed with primary metastatic breast cancer in 2012 at the age of 69 years. The primary tumor was strongly positive for the ER (> 80% of the cells with nuclear positivity) and HER2-negative. Metastatic lesions were detected in the lung and in the spine. Chemotherapy with Paclitaxel weekly was started, however after the first week the therapy was switched to the endocrine therapy with Letrozole based on the patient's wish.

### Sample preparation

Blood samples collected in EDTA tubes were processed within 2 hours. Blood samples collected in CellSave tubes were stored for 24–30 hours at room temperature before being processed. Mononuclear cells from both cancer patients’ and healthy donors’ blood spiked with SK-BR-3 cells were enriched by Ficoll density gradient centrifugation as previously described [34], fixed with 0.5% paraformaldehyde for 10min, and stained for keratins as described elsewhere [35].

Single cells were picked by micromanipulation (micro injector CellTramVario and micromanipulator TransferManNKII, Eppendorf Instruments, Hamburg, Germany) with the use of glass capillaries, allowing for the isolation of individual cells. Each individual cell was transferred in 1μl of PBS into the cap of a 0.2 ml PCR tube and stored at −80°C overnight. In order to obtain samples with pooled 3, 5 and 10 cells, every single cell was picked individually and transferred into a 0.2 ml PCR tube without touching the liquid already present in the tube, until the desired number of cells was riched.

FFPE SK-BR-3 material was cut in 5 μm thin sections and preprocessed as described before [36, 37]. Cross-links were removed by incubation of the slides in 1 M NaSCN at 56°C overnight. Subsequently, the slides were washed 3 × 3 min with TBS, stained with hematoxylin for 30s, rinsed with water, single cells were picked by micromanipulation.

### Whole genome amplification and quality control PCR

WGA was performed according to the manufacturers’ recommendations using 3 different kits: PCR-based Ampli1 (WG-001-050-R02, Silicon Biosystems), combined MDA-PCR PicoPlex (E2620L, New England Biolabs,), and MDA-based REPLI-g (150343, Qiagen) WGA kits. The WGA products after Ampli1 and PicoPlex underwent cleanup with NucleoSpin Gel and PCR Clean-up kit (740609, Macherey-Nagel). REPLI-g WGA products were cleaned according to the QIAGEN recommendations with ethanol for the FFPE samples and spin columns (51304, Qiagen) for the blood samples.

DNA concentration was measured with a Nanodrop1000 (Peqlab, Erlangen, Germany). Nanodrop was calibrated according to the manufacturer's recommendations using the CF-1 Calibration Fluid (CF1, ThermoFisher Scientific). The quality control of the WGA products was assessed by a multiplex PCR of the *GAPDH* gene as described elsewhere [36] with minor adaptations (Supplementary Material 1). Samples were considered of sufficient quality for further analyses if at least one of 200–400 bp bands was detectable.

### Next generation sequencing

Amplified DNA was investigated with whole exome sequencing on HiSeq2000 and IonProton platforms, unamplified DNA of the SK-BR-3 cells was sequenced with HiSeq2000. Sequence data are available at http://www.ebi.ac.uk/ena/data/view/PRJEB11307

### Data analysis

Raw data from the Ampli1- and PicoPlex-amplified samples underwent adapter clipping. PCR adapters of the Ampli1 kit are ligated to DNA sticky ends after MseI restriction of T^TAA sites [38], therefore adapters can be identified as oligonucleotide sequences framing TAA…(N)…T fragments. For PicoPlex-amplified samples we trimmed the first/last 14 bases as suggested by the manufacturer. Random hexamer primers of REPLI-g are complementary to the DNA and therefore did not need to be trimmed.

Further data analysis was done according to the GATK Best Practices recommendations [39, 40], detailed available as Supplementary Material 2. SNP/mutation and indel discovery was limited to protein coding exons only (downloaded from the CCDS Project database [41]).

Sensitivity, specificity, positive and negative predictive values of the variant calling analysis were evaluated based on the schema presented on Supplementary Figure S2. Calls from single cells (analyzed samples) were compared to calls made from unamplified DNA from bulk cell pellets (reference). Analyses were limited to SNP positions and alleles as defined in the dbSNP (Version 138) to minimize discrepancies from random error between samples. Truepositives (TP) are defined as known SNPs found in both the reference and analyzed samples. Falsepositives (FP) are known SNPs identified in analyzed samples but not present in reference. Conversely, known SNPs identified in the reference sample but not in the analyzed sample are falsenegatives (FN). Based on this definition, sensitivity (S), specificity (Sp), positive predictive value (PPV) and negative predictive value (NPV) were calculated as follows: S = TP/(TP + FN), Sp = TN/(TN + FP), PPV = TP/(TP + FP), NPV = TN/(TN + FN). Indel calling statistics were calculated similarly.

Venn diagrams were created with the used of BioVenn web application [42].

Allelic dropout (ADO) rate was calculated as follows: heterozygous SNPs in sample, present in reference, divided by their sum with homozygous SNPs in sample, present in reference as heterozygous SNPs.

CNAs were evaluated using Control-FREEC [43] with a window size of 30 kb, visualized and further analyzed using custom scripts (MATLAB R2015a, The MathWorks Inc.). Correlation among CNA profiles was calculated using Spearman correlation test.

## CONCLUSION

We comprehensively tested the effectiveness of WGA of single cells for exome sequencing by NGS. As an aspect of testing, we evaluated 3 WGA techniques, 2 NGS platforms, and the influence of material fixation for long term preservation. Although MDA-based WGA technique (REPLI-g kit) yielded the highest DNA amount, DNA quality was not adequate for SNP/mutation, indel, and CNA analysis.

PCR-based WGA technique (Ampli1 kit) combined with Illumina's HiSeq2000 platform demonstrated the best concordance with unamplified DNA for SNP/mutation and indel calling, both for EDTA- and CellSave-preserved cells with ADO rates 9–79%, mostly dependent on the amount of starting material. However, performance of the MDA-PCR combining WGA technique (PicoPlex kit) significantly improves with the number of pooled cells (increasing amount of input DNA), whereas performance of the PCR-based WGA technique did not significantly improve with increase of input material in any case.

The CNA profiles produced with MDA-PCR combining WGA technique on both HiSeq2000 and IonProton, independent of blood preservative, resembled unamplified DNA the most. Performance of CNA analysis of MDA-PCR combining WGA technique is not affected by input amount.

Our study shows the feasibility of genomic analysis of single cells isolated from differently preserved material, enabling advanced diagnostics such as on CTCs during cancer treatment for companion diagnostics.

## SUPPLEMENTARY MATERIALS FIGURES AND TABLES


