# Supplementary material for: Comparative study of whole genome amplification and next generation sequencing performance of single cancer cells
Source: Oncotarget. 2016 Jul 19;8(34):56066–80. doi: 10.18632/oncotarget.10701 (PMC5593545; doi:10.18632/oncotarget.10701)
Supplement: Supplementary file 1 [file oncotarget-08-56066-s001.pdf]

Comparative study of whole genome amplification and next generation sequencing performance of single cancer cells

Supplementary Materials

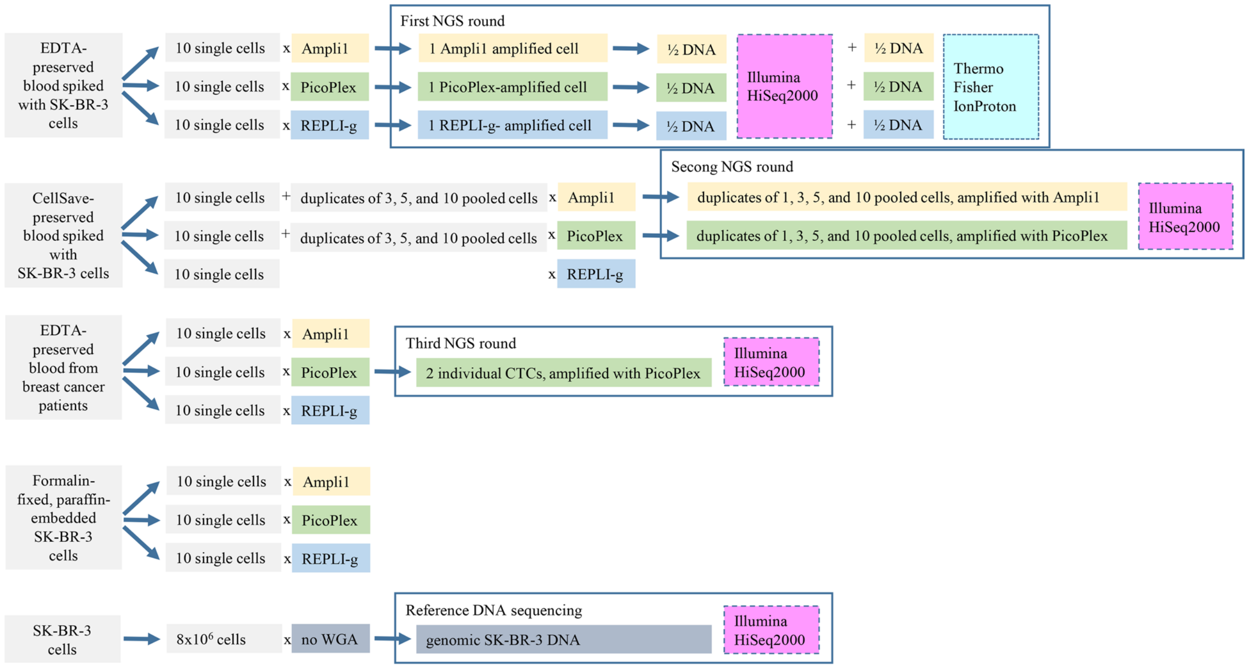

Supplementary Figure S1: Experimental design. SK-BR-3 breast cancer cell line cells were spiked into blood donors' blood, collected into EDTA- and CellSave tubes. Previously the same cell line cells were used to prepare formalin-fixed, paraffin-embedded material (FFPE). Blood from breast cancer patients was drawn into EDTA-tubes. Blood and FFPE samples were processed and used for picking of individual tumor cells: (**A**) 10 individual SK-BR-3 cells spiked and picked from EDTA-preserved blood; (**B**) 10 individual SK-BR-3 cells spiked and picked from CellSave-preserved blood; (**C**) 10 single SK-BR-3 cells picked from FFPE SK-BR-3 cells; and (**D**) 10 individual CTCs from breast cancer patients, from each group of samples. Collected cells were used for WGA with Ampli1, PicoPlex, and REPLI-g WGA kits. For the EDTA-preserved SK-BR-3 cells, 3 representative whole genome amplified cells, one per WGA kit, underwent NGS on both HiSeq200 and IonProton platforms. Taking SNP, indel, and CNA results into consideration, next NGS round on HiSeq2000 included CellSave-preserved SK-BR-3 cells, amplified with Ampli1 and PicoPlex, and patients CTCs, amplified with PicoPlex. Unamplified SK-BR-3 DNA from unfixed cells was sequenced on HiSeq2000.

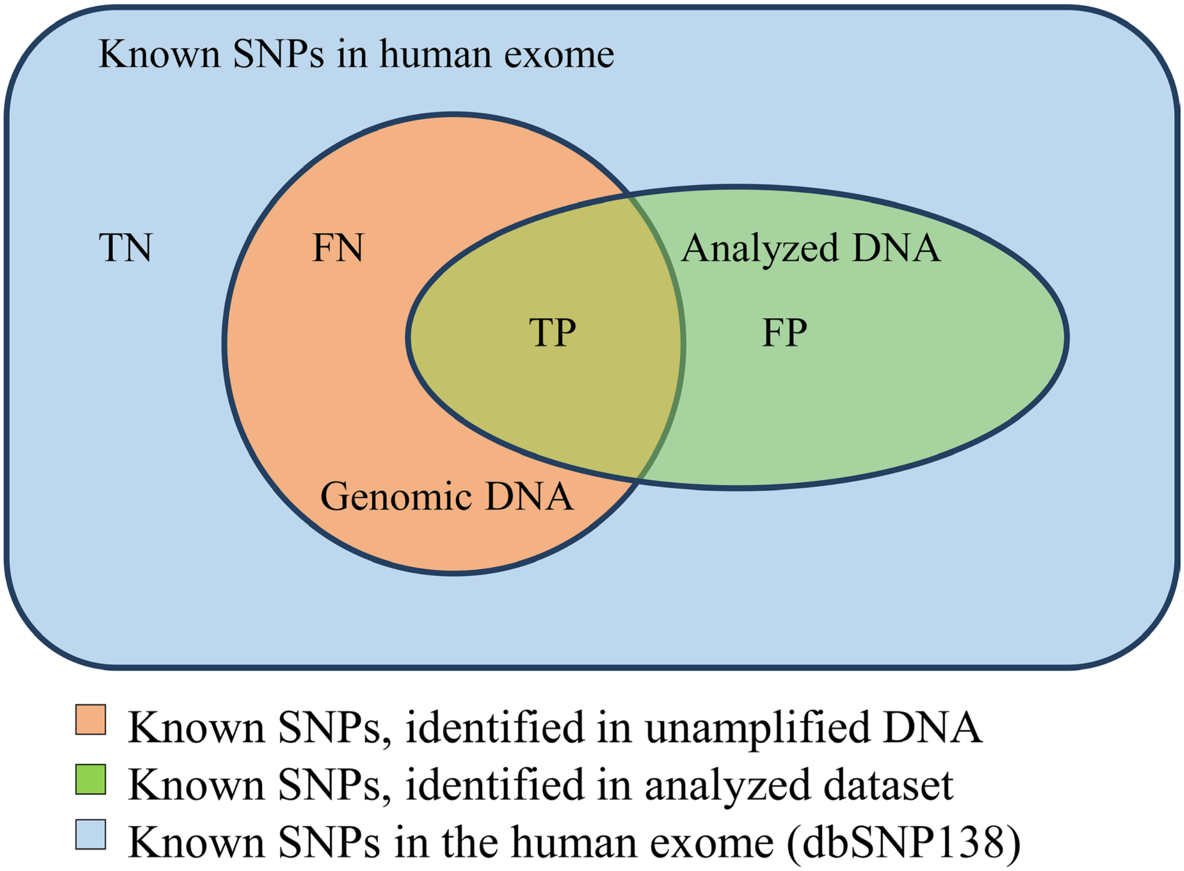

Supplementary Figure S2: Definition of SNP calls, identified in analyzed dataset in comparison to reference as true-positive, false-positive, true-negative, and false-negative SNPs. TP – true-positive calls; FP – false-positive calls; TN – true-negative calls; FN – false-negative calls.

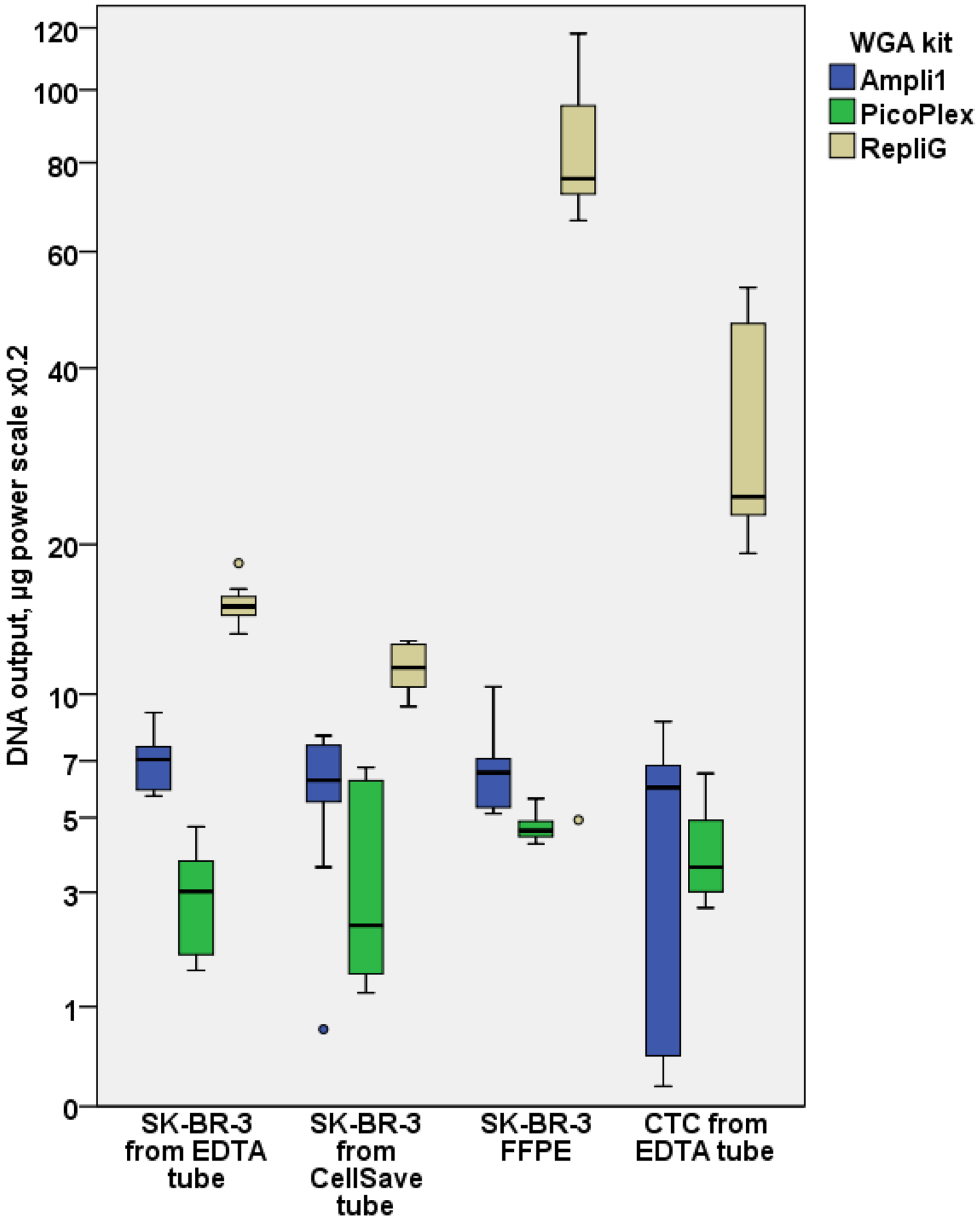

Supplementary Figure S3: DNA yield in respect to WGA kit in groups of single SK-BR-3 cells, picked from EDTA- and CellSave preserved blood, FFPE material, and CTCs, picked from EDTA-preserved blood of breast cancer patients.

SUPPLEMENTARY MATERIAL 1

The quality of the WGA products was assessed by a multiplex PCR of the GAPDH gene producing fragments of 100, 200, 300, and 400 bp fragments from non-overlapping target sites as described elsewhere [1]. Since the original 200 bp fragment is not amplified by the Ampli1 WGA kit, we used the following primers to produce a 200 bp fragment: fw: 5′-AAGATCATCAGGTGAGGAAGGC-3′ rev: 5′-CCCCAGCTCTCATACCATGAGTC-3′. The 5′–3′ primers for the 100, 300, and 400 bp fragments were as follows: 100fw gttccaatatgattccaccc; 100rev ctcctggaagatggtgatgg; 300fw aggtgagacattcttgctgg; 300rev tccactaaccagtcagcgtc; 400fw acagtccatgccatcactgc and 400rev gcttgacaaagtggtcgttg

PCR conditions were optimized for a reaction of 15 µl total volume with input of 100 ng DNA as follows: 0.75 U AmpliTaq Gold DNA Polymerase (Applied Biosystems, 4486226), 0.2 mM of each ATP, GTP, CTP, TTP; 0.136 µM of each primer, and 2 mM $MgCl_2$ (Applied Biosystems, R01911). Human leukocyte DNA was used as positive control for the multiplex PCR. The PCR program was as follows: 95°C for 5 min; 35 cycles of 94°C for 30 sec, 64°C for 30 sec, 72°C for 45 sec; final elongation at 72°C for 7 min. PCR was conducted with the use of the peqSTAR 96X Thermocycler (VWR, Darmstadt, Germany).

PCR products were analyzed in a 2% agarose TAE ethidium bromide-stained gel. Samples were considered to be of sufficient quality for further analyses if at least one of the 200, 300, and 400 bp bands was detected.

REFERENCES

1. van Beers EH, Joosse SA, Ligtenberg MJ, Fles R, Hogervorst FB, Verhoef S and Nederlof PM. A multiplex PCR predictor for aCGH success of FFPE samples. Br J Cancer. 2006; 94:333–337.

SUPPLEMENTARY MATERIAL 2

Data analysis was performed according to the GATK Best Practices recommendations [1, 2]. Exome capturing was performed with “BGI Exome Enrichment Kit (59M) and Capture” for sequencing on HiSeq2000 and “Ion AmpliSeq exome RDY kit” for sequencing on IonProton. The corresponding exome regions were used respectively for calculation of descriptive statistics over target regions and during post-alignment data processing. To ensure the location of made calls within the exome and to unify results of SNP and indel calling between the datasets, SNP/mutation and indel discovery was limited to protein coding exons only (downloaded from the CCDS Project database [3, 4]).

Reference datasets used for the analysis

Human genome UCSC hg19	[5]
dbsnp_138.hg19.vcf	[6]
Mills_and_1000G_gold_standard.indels.hg19.sites.vcf	[7]
UCSC_CCDS_per_exon.bed	[4]
HG19 snpEff database	[8]
control file for FREEC was generated out of alignment of 185 reference European female genomes, obtained from 1000 Genome database	[9]
GEM_mapp_hg19/out100m1_hg19.gem	[10]
COSMIC database	[11, 12]

Programs

bwa mem	[13]
gatk	[14]
picard	[15]
samtools	[16]
trimmomatic	[17]
snpEff	[18]
snpSift	[19]
Control-FREEC	[20, 21]

REFERENCES

1. DePristo MA, Banks E, Poplin R, Garimella KV, Maguire JR, Hartl C, Philippakis AA, del Angel G, Rivas MA, Hanna M, McKenna A, Fennell TJ, Kernytsky AM, et al. A framework for variation discovery and genotyping using next-generation DNA sequencing data. Nat Genet. 2011; 43:491–498.
2. Van der Auwera GA, Carneiro MO, Hartl C, Poplin R, Del Angel G, Levy-Moonshine A, Jordan T, Shakir K, Roazen D, Thibault J, Banks E, Garimella KV, Altshuler D, et al. From FastQ data to high confidence variant calls: the Genome Analysis Toolkit best practices pipeline. Curr Protoc Bioinformatics. 2013; 11:11 10 11–11 10 33.
3. Farrell CM, O’Leary NA, Harte RA, Loveland JE, Wilming LG, Wallin C, Diekhans M, Barrell D, Searle SM, Aken B, Hiatt SM, Frankish A, Suner MM, et al. Current status and new features of the Consensus Coding Sequence database. Nucleic Acids Res. 2014; 42:D865–872.
4. CCDS Project. Available from: http://www.ncbi.nlm.nih.gov/projects/CCDS/CcdsBrowse.cgi.
5. Human genome HG19 Available from: http://hgdownload.soe.ucsc.edu/goldenPath/hg19/bigZips/.
6. Database of Single Nucleotide Polymorphisms (dbSNP). Bethesda (MD): National Center for Biotechnology Information, National Library of Medicine. (dbSNP Build ID: 137). Available from: http://www.ncbi.nlm.nih.gov/SNP/.
7. Mills and 1000G gold standard indels. Available from: ftp://ftp.broadinstitute.org/bundle/2.5/hg19/.

8. HG19 snpEff database. Available from: http://snpeff.sourceforge.net/download.html#databases.
9. Genomes Project C, Abecasis GR, Auton A, Brooks LD, DePristo MA, Durbin RM, Handsaker RE, Kang HM, Marth GT, McVean GA. An integrated map of genetic variation from 1,092 human genomes. Nature. 2012; 491:56–65.
10. GEM_mapp_hg19/out100m1_hg19.gem for Control-FREEC.
11. COSMIC database. Available from: cancer.sanger.ac.uk.
12. Forbes SA, Beare D, Gunasekaran P, Leung K, Bindal N, Boutselakis H, Ding M, Bamford S, Cole C, Ward S, Kok CY, Jia M, De T, et al. COSMIC: exploring the world's knowledge of somatic mutations in human cancer. Nucleic Acids Res. 2015; 43:D805–811.
13. Li H. Aligning sequence reads, clone sequences and assembly contigs with BWA-MEM. 2013.
14. McKenna A, Hanna M, Banks E, Sivachenko A, Cibulskis K, Kernytsky A, Garimella K, Altshuler D, Gabriel S, Daly M, DePristo MA. The Genome Analysis Toolkit: a MapReduce framework for analyzing next-generation DNA sequencing data. Genome Res. 2010; 20:1297–1303.
15. Picard tools. Available from: http://picard.sourceforge.net.
16. Li H, Handsaker B, Wysoker A, Fennell T, Ruan J, Homer N, Marth G, Abecasis G, Durbin R, Genome Project Data Processing S. The Sequence Alignment/Map format and SAMtools. Bioinformatics. 2009; 25:2078–2079.
17. Bolger AM, Lohse M, Usadel B. Trimmomatic: a flexible trimmer for Illumina sequence data. Bioinformatics. 2014; 30:2114–2120.
18. Cingolani P, Platts A, Wang le L, Coon M, Nguyen T, Wang L, Land SJ, Lu X, Ruden DM. A program for annotating and predicting the effects of single nucleotide polymorphisms, SnpEff: SNPs in the genome of Drosophila melanogaster strain w1118; iso-2; iso-3. Fly. 2012; 6:80–92.
19. Cingolani P, Patel VM, Coon M, Nguyen T, Land SJ, Ruden DM, Lu X. Using Drosophila melanogaster as a Model for Genotoxic Chemical Mutational Studies with a New Program, SnpSift. Front Genet. 2012; 3:35.
20. Boeva V, Popova T, Bleakley K, Chiche P, Cappo J, Schleiermacher G, Janoueix-Lerosey I, Delattre O, Barillot E. Control-FREEC: a tool for assessing copy number and allelic content using next-generation sequencing data. Bioinformatics. 2012; 28:423–425.
21. Boeva V, Zinovyev A, Bleakley K, Vert JP, Janoueix-Lerosey I, Delattre O, Barillot E. Control-free calling of copy number alterations in deep-sequencing data using GC-content normalization. Bioinformatics. 2011; 27:268–269.

	Program and command	Specifications or differing from default parameters	References
1A. PREPROCESSING AND ALIGNMENT FOR PAIRED-END HISEQ2000 READS			
Clip WGA adapters if present		For Ampli1 and PicoPlex amplified samples	
Trim	trimmomatic	PE ILLUMINACLIP:2:30:10 LEADING:3 TRAILING:3 SLIDINGWINDOW:4:10 MINLEN:25 TOPHRED33	
Align to the genome	bwa mem	-t 30 -v 0 -M -R	UCSC hg19
1B. PREPROCESSING AND ALIGNMENT FOR SINGLE-END IONPROTON READS			
Sort and convert bam file to fastq	samtools sort<br>samtools bam2fq	-n<br>-n -O -s	
Clip WGA adapters if present		For Ampli1 and PicoPlex amplified samples	
Trim	trimmomatic	SE LEADING:3 TRAILING:3 SLIDINGWINDOW:4:10 MINLEN:25 TOPHRED33	
Align to the genome	bwa mem	-t 30 -v 0 -M -R	UCSC hg19
2. POSTALIGNMENT PROCESSING			
Sort sam file and convert to bam	picard SortSam	SORT_ORDER=coordinate VERBOSITY=ERROR COMPRESSION_LEVEL=0	
Mark duplicates	picard MarkDuplicates	VERBOSITY=ERROR COMPRESSION_LEVEL=0	
Index bam file	samtools index		
Realign indels	gatk RealignerTarget Creator	-nt 24	UCSC hg19<br>Mills_and_1000G_gold_standard.indels.hg19.sites.vcf
	gatk IndelRealigner	-compress 0 -model USE_READS -LOD 0.4	UCSC hg19<br>Mills_and_1000G_gold_standard.indels.hg19.sites.vcf
Recalibrate bases	gatk BaseRecalibrator	-nct 24	UCSC hg19<br>dbsnp_138.hg19.vcf<br>Mills_and_1000G_gold_standard.indels.hg19.sites.vcf
	gatk PrintReads	-BQSR -compress 0	UCSC hg19
3. DISCOVER SNPS AND INDELS			
SNP and indel calling	gatk HaplotypeCaller	-stand_call_conf 30 -stand_emit_conf 30 -gt_mode DISCOVERY -out_mode EMIT_ALL_CONFIDENT_SITES -ploidy 3<br>--annotation FisherStrand --annotation QualByDepth --annotation HaplotypeScore --annotation HomopolymerRun --annotation RMSMappingQuality --annotation ReadPosRankSumTest	UCSC hg19<br>dbsnp_138.hg19.vcf<br>UCSC_CCDS_per_exon.bed
Select for SNPs	gatk SelectVariants	-selectType SNP	UCSC hg19
Annotate HRun	gatk VariantAnnotator	--annotation HomopolymerRun	UCSC hg19<br>dbsnp_138.hg19.vcf
Filter for quality and GQ	snpSift filter	(QD >= 5) & (MQ > 25) & (QUAL > 30) & (FS < 60) & (SOR < 4) & (HRun < 5) & (GEN[*].GQ >= 20)	
Annotate with snpEff	snpEff		HG19 snpEff database
Select for INDELs	gatk SelectVariants	-selectType INDEL	UCSC hg19
Filter for quality and GQ	snpSift filter	(QD >= 2) & (MQ > 25) & (QUAL > 20) & (FS < 200) & (SOR < 10) & (GEN[*].GQ >= 20)	
Annotate with snpEff	snpEff		HG19 snpEff database
investigate mutations (for patient's data only)	gatk VariantAnnotator	#Annotate with COSMIC data<br>-comp:COSMIC #{Cosmic} -resource #{Cosmic}	hg19_cosmic_v54_120711 (#{Cosmic})
4. COPY NUMBER ANALYSIS			
Create mpileup file	samtools mpileup	-E	
Run Control-FREEC	freec	breakPointType = 4, forceGCcontentNormalization = 2, noisyData = TRUE, ploidy = 3 (for SK-BR-3, and ploidy = 2 for CTCs), printNA = FALSE, readCountThreshold = 50, sex = XX, window = 30000, uniqueMatch = TRUE	UCSC hg19<br>GEM_mapp_hg19/out100m1_hg19.gem<br>control file for FREEC for SK-BR-3 analysis, no control for CTCs
